# Supplementary material for: Biomechanical Role of Epsin in Influenza A Virus Entry
Source: Membranes (Basel). 2022 Sep 5;12(9):859. doi: 10.3390/membranes12090859 (PMC9505878; doi:10.3390/membranes12090859)
Supplement: Supplementary file 1 [file membranes-12-00859-s001.zip › membranes-1869166-supplementary.pdf]

Supplementary Materials for

Biomechanical Role of Epsin in Influenza A Virus Entry

Jophin G. Joseph[1,†], Rajat Mudgal[2], Shan-Shan Lin[1], Akira Ono[2], Allen P. Liu[1,3,4,5,*]

[1] Department of Mechanical Engineering, University of Michigan, Ann Arbor, Michigan, 48109, USA

[2] Department of Microbiology and Immunology, University of Michigan, Ann Arbor, Michigan, 48109, USA

[3] Department of Biomedical Engineering, University of Michigan, Ann Arbor, Michigan, 48109, USA

[4] Cellular and Molecular Biology Program, University of Michigan, Ann Arbor, Michigan, 48109, USA

[5] Department of Biophysics, University of Michigan, Ann Arbor, Michigan, 48109, USA

[*] Correspondence: A.P.L.: allenliu@umich.edu

†Current position: Senior Engineer, Zoetis Inc., Kalamazoo, Michigan, USA

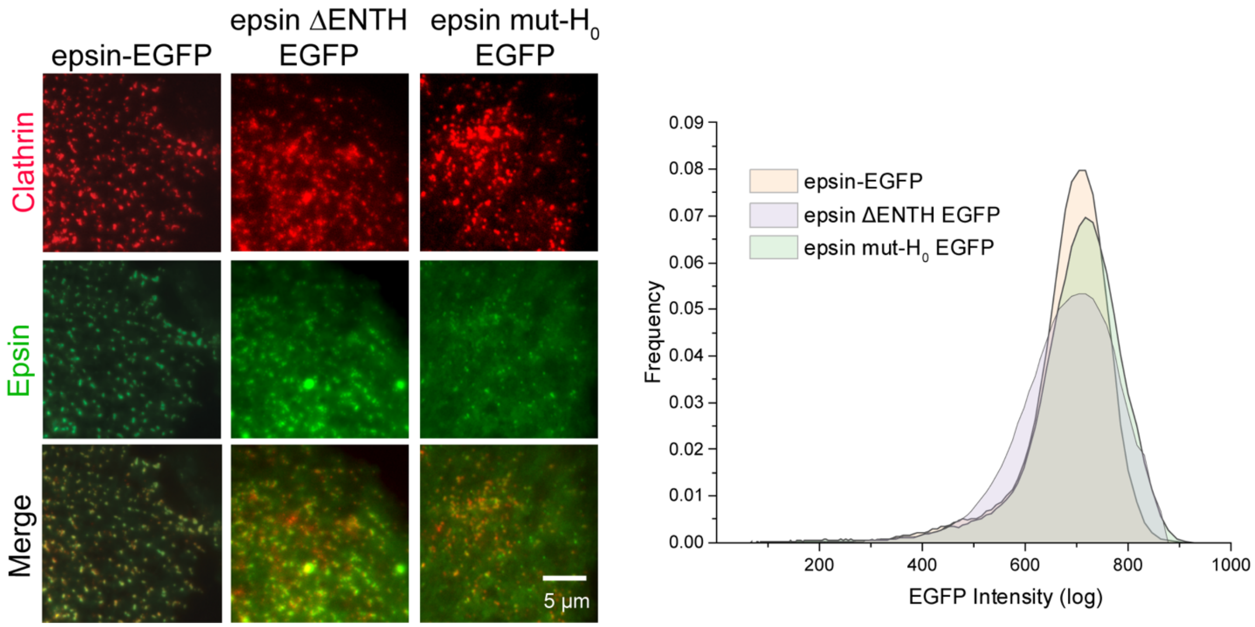

Supplementary Figure S1. Expression levels of different epsin constructs. a. Representative fluorescence images of stable RPE cells expressing mCherry-Clc and different EGFP tagged epsin constructs (left). Flow cytometry analysis shows the stable cell line have similar expression levels of EGFP-tagged epsin constructs (right).

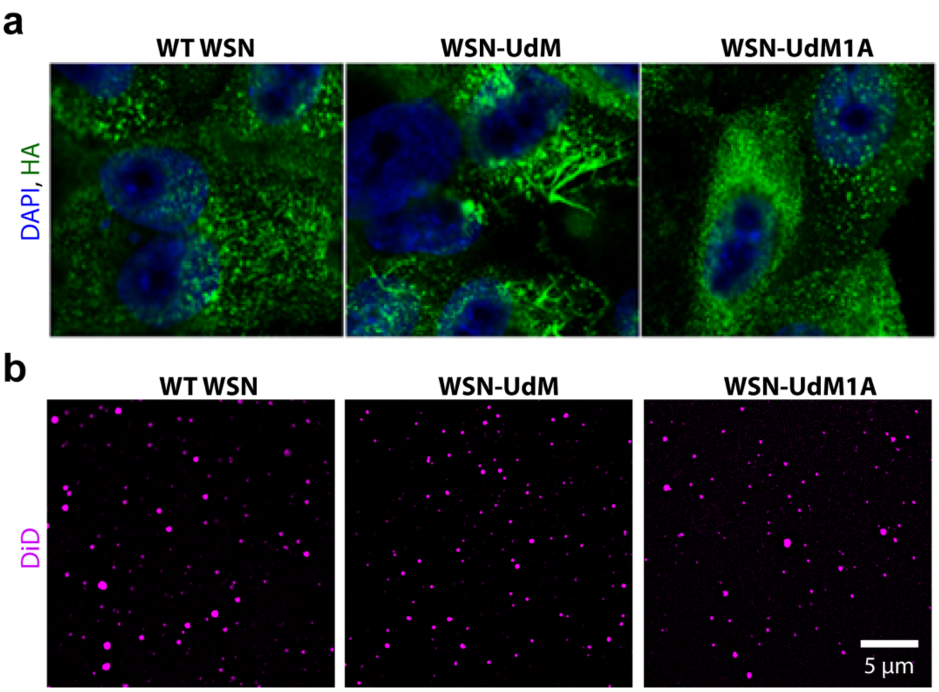

Supplementary Figure S 2. Spherical and filamentous IAV morphologies and labeling. (**a**). MDCK cells producing spherical (WSN WT), mixed population of filamentous and spherical (WSN-UdM) and isogenic spherical (WSN UdM1A) IAVs. Signal are detected by immunostaining for HA (non-permeabilized). (**b**). Harvested WSN WT, WSN-UdM and WSN-UdM1A IAV particles labeled with DiD lipophilic dye and imaged by fluorescence confocal microscopy. Note that the WSN-UdM particles do not appear to be filamentous as they can be diffraction-limited, but they are filament-forming particles.

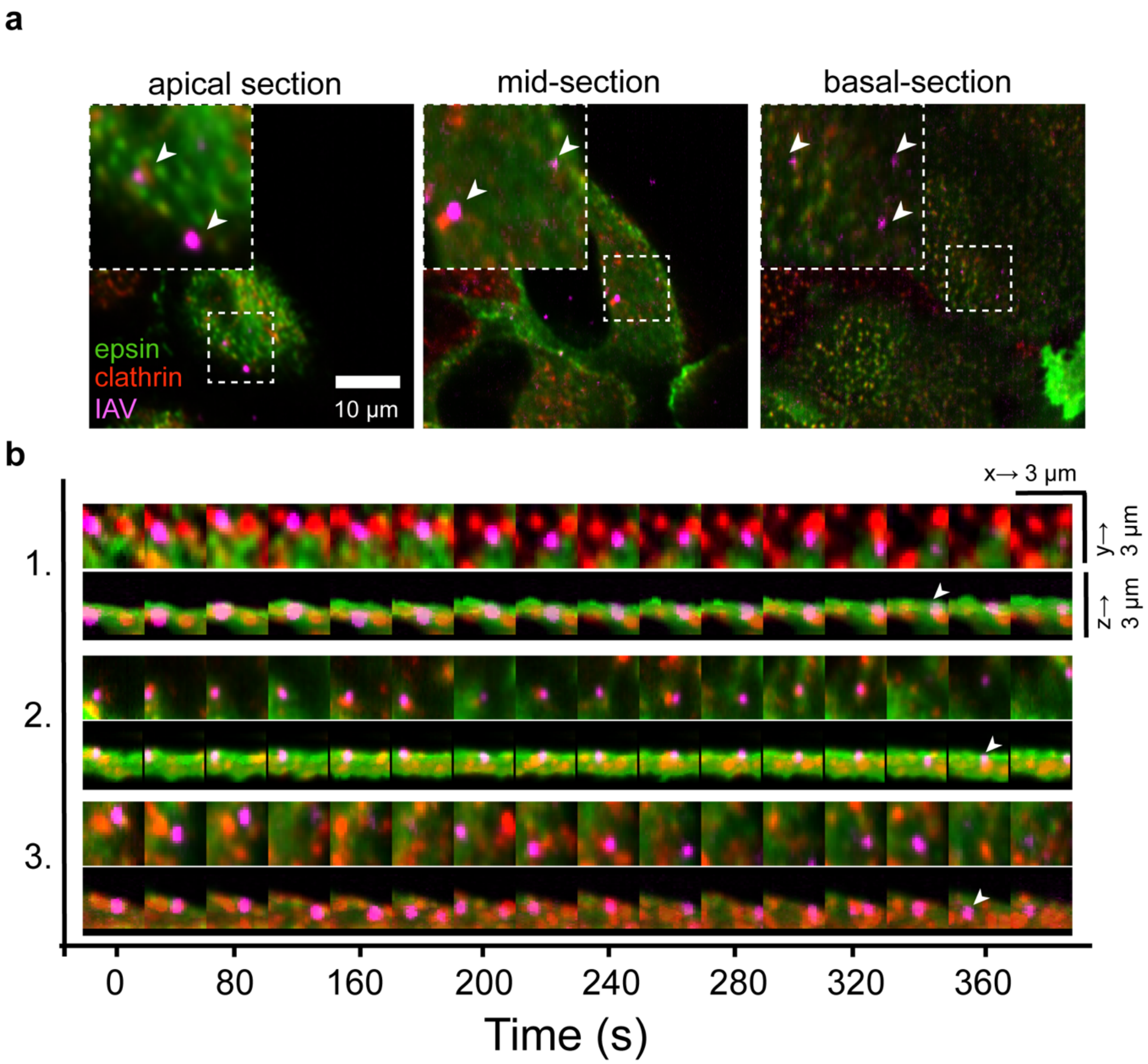

Supplementary Figure S 3. IAV internalization visualized using lattice light sheet microscopy. (**a**)**.** Different sections of RPE cells overexpressing epsin-EGFP and mCherry-Clc. IAVs are shown with white arrows (inset). (**b**)**.** Three example trajectories of IAV (magenta) internalization in *x-y* and *x-z* spatial orientations. The fully internalized IAV is shown using white arrows.

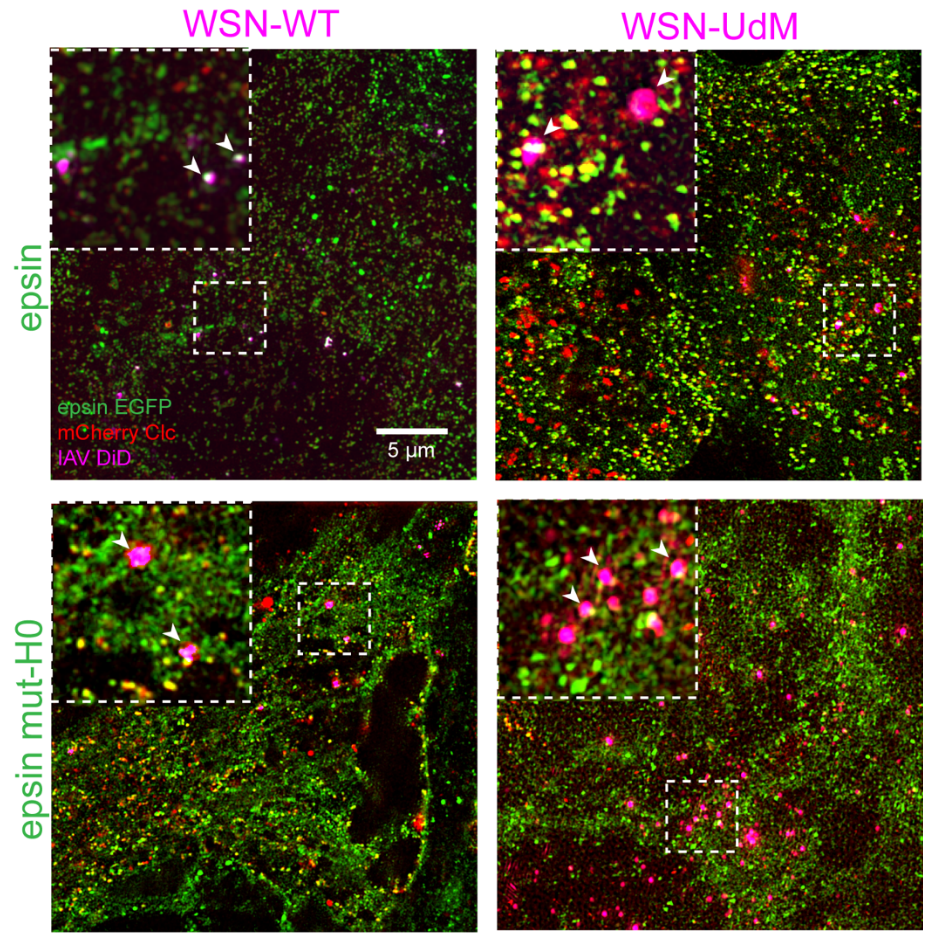

Supplementary Figure S4. Colocalization of spherical and filament-forming IAVs to CCSs. 3D SIM images (maximum intensity projection) of spherical (WSN-WT) and filament-forming (WSN-UdM) viruses bound to CCSs in apical section of RPE cells stably expressing epsin EGFP or epsin mut-H_0 EGFP and mCherry-Clc. White arrows in the inset show IAVs bound to CCSs on the cell surface.

Supplementary Table S1. Comparison of viral infectivity with or without DiD labeling. MDCK cells were infected with IAVs with or without DiD labeling. Virus titers (PFU/mL) were quantified by plaque assay. Data are representative of the mean ± SD from two replicates.

Viral strain	DiD (-)	DiD (+)
**WSN-WT**		$2.00 \pm 0.14 \times 10^{8}$
**WSN-UdM**		$1.30 \pm 0.42 \times 10^{7}$
**WSN-UdM-1A**		$1.15 \pm 0.21 \times 10^{7}$
